# Overlapping pathways of migraine and the endocannabinoid system: Potential therapeutic targets

**DOI:** 10.1016/j.neurot.2026.e00833

**Published:** 2026-01-17

**Authors:** Adriana Della Pietra, Andrew F. Russo

**Affiliations:** a Department of Molecular Physiology and Biophysics, University of Iowa, Iowa City, IA, USA; b Department of Neurology, University of Iowa, Iowa City, IA, USA; c Veterans Affairs Healthcare System, Iowa City, IA, USA

**Keywords:** 2-AG, Anandamide, Analgesic, Circadian rhythm, Glymphatic clearance

## Abstract

Migraine is a disabling neurovascular disorder with limited therapeutic options, especially for patients unresponsive to current treatments targeting calcitonin gene-related peptide (CGRP) signaling. The endocannabinoid system (ECS) has emerged as a promising alternative for migraine modulation, offering analgesic, anti-inflammatory, and neuroimmune-regulatory effects through its main ligands, anandamide (AEA) and 2-arachidonoylglycerol (2-AG), and their degrading enzymes. This review provides an updated map of endocannabinoid components in central and peripheral migraine-relevant regions, highlighting their spatial distribution and functional regulation in animal models. We summarize the available preclinical evidence supporting the anti-nociceptive effects of endocannabinoid-degrading enzyme inhibitors and cannabinoid receptor agonists/antagonists, with particular emphasis on the therapeutic potential of multi-target compounds. Moreover, we explore non-canonical ECS pathways, including TRPV1, D2 dopamine receptors, serotonergic and ion channel interactions, and their roles in modulating CGRP release and trigeminovascular signaling to treat migraine pathophysiology. Finally, we propose two sleep-related directions for treatments involving ECS modulation of circadian rhythms and glymphatic clearance. Although human translational data are limited, the ECS offers a multifaceted framework for developing next-generation therapeutics targeting migraine pathophysiology.

## Introduction

Migraine is not simply a headache. It is a multifaceted neurovascular disease, affecting up to 14 % of people worldwide [1]. As such, migraine represents a major social and economic burden [2,3]. It is the world's second leading cause of disability regardless of age and gender [4]. Notably, it scores as the major life impairing disease in young women [5]. While current treatment options can alleviate migraine pain, many patients are not helped or experience side effects. Even the recently developed CGRP-based drugs leave roughly 30–50 % of migraine patients untreated [6].

As alternative treatments, instead of using highly addictive painkillers, such as opioids, there is a great need for alternative, effective and safe medications. Despite potential negative psychoactive side effects and potential medication-overuse headache risk [7,8], medical marijuana has been shown to decrease the frequency of migraine headaches [9], suggesting a perspective for cannabinoids in treating migraine. The use of cannabis to relieve pain dates to ancient civilizations [10]. Cannabis has been used for millennia in ayurvedic medicine for the management of pain, nausea, and anxiety, as well as to promote appetite and sleep [10]. The cannabis plant has complex natural compounds, including, in addition to the generally safe cannabidiol (CBD), the psychotropic delta9-tetrahydrocannabinol (THC), and dozens of poorly characterized components [11]. Recent studies have pointed to the therapeutic potential of a combination of CBD and THC in preclinical migraine models [12,13] and in a clinical trial [14,15]. These CBD and THC findings point to the importance of targeting the body's endocannabinoid system (ECS) [16,17]. Thus, the stage is set for testing the enhancement of ECS signaling pathways that might provide analgesia with minimal adverse effects.

Endocannabinoids are known for their anti-nociceptive signaling via the ECS and as such represent an attractive alternative to psychotropic components of phyto-cannabinoids [18]. Nociceptive signaling in migraine pain areas is sensitive to anti-nociceptive endocannabinoids, which will be a focus of this review. The two main endocannabinoids are 2-arachidonoylglycerol (2-AG) and anandamide (AEA). The anti-nociceptive efficiency of 2-AG and AEA is determined by their enzymatic synthesis, activity on cannabinoid receptors type 1 and 2 (CB1 and CB2), and degradation. 2-AG is synthesized and released on-demand when phospholipase C (PLC) acts on lipid precursors in cells containing the synthesizing enzyme diacylglycerol lipase (DAGL). DAGL converts PLC-derived diacylglycerol into 2-AG, or into another monoacylglycerol such as 2-oleoylglycerol [19]. 2-AG acts as a full agonist at both CB1 and CB2 receptor types [20]. 2-AG is hydrolyzed by monoacylglycerol lipase (MAGL) and α/β -hydrolase domain protein 6 (ABDH6). The endocannabinoid AEA and other N-acyl ethanolamines, including palmitoylethanolamide (PEA) and oleoylethanolamide (OEA), are formed from N-acyl-phosphatidylethanolamine (NAPE) through the action of the enzyme N-acylphosphatidylethanolamine-specific phospholipase D (NAPE-PLD) [21]. AEA is a partial agonist at CB1 and CB2 receptors [20]. AEA is hydrolyzed by fatty acid amide hydrolase (FAAH) [22,23]. Although direct evidence is still lacking that boosting endocannabinoid synthesis can produce analgesic effects, there has been recent progress in the development of selective and potent MAGL and FAAH inhibitors with the aim of achieving migraine pain relief [24]. MAGL and FAAH inhibitors provide excellent starting points for enhancing endocannabinoids activity. Indeed, a key point is that the endocannabinoids and their enzymes are distinctly active in different peripheral and central migraine pain signaling areas [25,26]. Therefore, to achieve an effect in all these areas, inhibition of at least FAAH and MAGL would be ideal.

In this review, we will show the overlap between the ECS and migraine-related signaling areas within the CNS and PNS. We will also discuss current strategies under preclinical investigation to treat migraine by targeting endocannabinoid-degrading enzymes, CB1/2 receptors, and other associated receptors, ion channels, and mechanisms.

## Endocannabinoid system in migraine pathways

To exploit the endocannabinoid system to achieve migraine pain analgesia, the components of the endocannabinoid system (synthesizing enzymes, CB1/2 receptors, the endocannabinoids, and degrading enzymes) have to be expressed and active in areas mediating migraine pathophysiology. We compiled available evidence on the expression and/or activity of ECS components in migraine-relevant brain regions. The information was drawn from multiple sources, not limited to migraine-specific literature. It includes data from diverse experimental models, including neuropathic, osteoarthritis pain, stress and other models. From the control groups in these studies, basal levels and/or activity of CB1/CB2, AEA, 2-AG, and MAGL/FAAH in specific brain regions were extrapolated. All the regions mentioned are known to be involved in migraine signaling. To date there has been a paucity of studies on the endocannabinoid synthesizing enzymes with respect to migraine, so we will begin with the receptors.

### CB1 and CB2 receptor localization in migraine-relevant areas

**Fig. 1:**
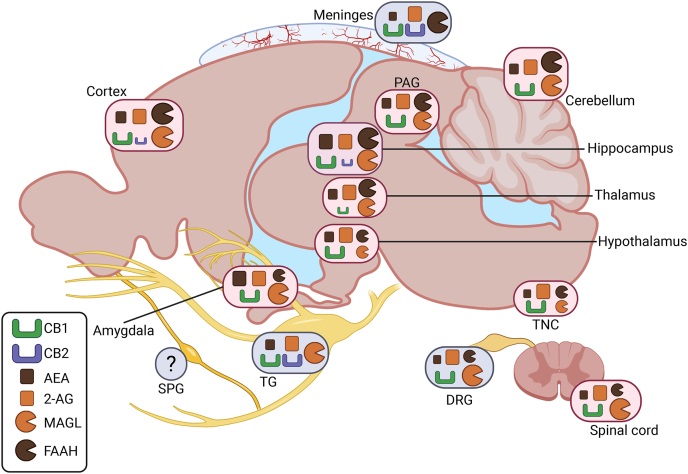
**Sites of AEA, 2-AG, their receptors CB1/2, and their main degrading enzymes MAGL and FAAH in rodent CNS and PNS areas associated with migraine signaling.** Both cannabinoid receptor type 1 and 2 (CB1 and CB2) are expressed in the cortex, hippocampus, meninges, and trigeminal ganglion (TG), although CB2 expression is generally much lower than CB1 in the CNS compared to peripheral tissues. The endocannabinoids 2-arachidonoylglycerol (2-AG) and anandamide (AEA) are present in all regions shown, with 2-AG typically more abundant than AEA, except in the amygdala and hippocampus where levels are comparable. The degrading enzymes monoacylglycerol lipase (MAGL) and fatty acid amide hydrolase (FAAH) exhibit high and comparable expression in the cortex, periaqueductal gray (PAG), cerebellum, hippocampus, and thalamus. In contrast, their levels are low in the hypothalamus and brainstem. MAGL predominates over FAAH in the spinal cord, amygdala, dorsal root ganglia (DRG), and is exclusively expressed in the TG. FAAH, conversely, is uniquely present in the meninges. The expression and activity of the endocannabinoid system components in the sphenopalatine ganglia (SPG) is currently unknown as indicated by the question mark. Peripheral cranial structures are highlighted with a blue oval, while central nervous system regions are in pink. The relative abundance of each endocannabinoid system component is represented by the proportional size of its symbol. Although comparative data exist for other species, this figure focuses on the murine models, where most of the current evidence listed in section Endocannabinoid system in migraine pathways is available.

CB1 receptors, the first identified cannabinoid receptors, are widely distributed in both neurons and glia of many central and peripheral nervous system (CNS and PNS) regions involved in pain modulation and migraine [27,28] (Fig. 1).

Functionally, CB1 receptors are enriched at presynaptic terminals and axons, where they regulate synaptic transmission [29,30]. They are present in the rat trigeminal ganglia [31], rat and human dorsal root ganglia [32,33], and dermal sensory nerve endings, where they modulate afferent pain signaling [34]. From here, CB1 are importantly expressed in the rat brainstem trigeminal nucleus caudalis (TNC) [27], which collects and further transmits the incoming nociceptive signals from the peripheral sites to the thalamus, and in the rat periaqueductal gray (PAG), whose functional connectivity has been connected to brain hyperexcitability in migraine [27]. Overall, CB1 receptors are the most abundant G protein–coupled receptors in both rat and human brain [35]. There is also fairly high expression in many other areas in the rat brain, including the cerebral cortex, hippocampus, and cerebellum, and moderate to low levels in the amygdala, hypothalamus, thalamus, and spinal cord [36,37].

In the context of migraine, all these regions have key functions. The cerebral cortex is involved in the process of cortical spreading depolarization (CSD), the electrophysiological mechanism underlying aura, and with the amygdala contributes to pain processing and emotional perception of migraine [38,39]. Hippocampal structural and functional alterations have been reported in migraine patients and are associated with disease progression [40]. The thalamus, hypothalamus, and cerebellum play key roles in migraine-related hypersensitivity, including allodynia and photophobia [[41], [42], [43]]. Finally, the spinal cord and dorsal root ganglia (DRG) are integral to peripheral sensory transduction and modulation [44], including nociceptive signaling relevant to migraine pain. Comparison with human data from quantitative autoradiographic studies indicates that most CB1 expression patterns are broadly translatable across species [35]. However, in humans, the amygdala displays some of the highest receptor densities, higher than the moderate levels typically reported in rats [35].

In contrast, CB2 receptors, mostly studied in rodent or cell-culture models, were initially identified in splenic macrophages [45]. These receptors are predominantly expressed in murine meningeal immune cells and only sparsely in CNS (Fig. 1). The actions of cannabinoids in specific dural immune cells through CB1/2 receptors in relationship to migraine has been already widely discussed by us [46]. Additionally, cerebrovascular endothelial cells exhibit CB2 receptor expression alongside CB1 and the AEA non-conventional TRPV1 receptors [47,48]. Interestingly, recent studies show CB2 receptors expression in microglia and, to a lesser extent, in neurons within the prefrontal cortex and hippocampal CA2/3 regions [49,50]. In neurons, CB2 receptors influence inhibitory plasticity, gamma oscillations, and excitability, acting independently of CB1 receptors [51,52]. In microglia, CB2 receptors modulate their activation and induced neuroinflammation [53,54], mechanisms relevant to migraine chronification and other pain disorders. Glia actively participate in immune functions [55], including the release of pro-inflammatory cytokines and facilitation of central sensitization [56], processes that contribute to the initiation and maintenance of migraine pain. Unlike microglia, the presence of CB2 receptors on astrocytes, regulating the glymphatic function, a process increasingly implicated in migraine pathophysiology [57], remains debated. Although some studies report CB2 in astrocytic cultures [58,59], these findings may be driven by artificial culture conditions [60]. Most evidence suggests that CB2 is absent in astrocytes under physiological conditions [61,62]. Although their expression is relatively limited, CB2 receptors are increasingly recognized as key modulators of nociception and neuroimmune signaling [63], positioning them as much as CB1 receptors as promising therapeutic targets for migraine and other pain conditions.

### Expression of AEA and 2-AG in migraine-relevant areas

Endocannabinoids are expressed in several migraine-relevant regions of the brain and trigeminovascular system (Fig. 1). In general, 2-AG levels tend to be higher than AEA. In human and rat meninges [64] and rat TG [65,66], basal levels of 2-AG are higher than AEA. While the role of DRG in migraine is less appreciated than the TG, basal 2-AG levels also appear higher than AEA in rat DRG, as quantified in the control group of neuropathic pain models [67]. Similarly, there is higher levels of 2-AG compared to AEA in other rat CNS regions related to migraine signaling. These regions include the TNC [65,66], PAG [68], spinal cord [65,66], cerebellum, hypothalamus [69], and thalamus [70] (Fig. 1). Basal endocannabinoid levels in the PAG [68] and thalamus [70] were extrapolated from neuropathic pain studies, while hypothalamic levels were taken from control groups in a study examining endocannabinoid changes related to fasting, feeding, and satiation [69]. In the rats and mice amygdala, which may be involved in affective aspects of migraine pain, 2-AG and AEA levels are comparable, as observed in control groups versus stress and osteoarthritis pain models [71,72]. There are some species differences in relative levels, for example, the hippocampus in mice has comparable levels of AEA and 2-AG [71,72], while in the rat 2-AG is higher than AEA [73]. A caveat to these measurements is that they do not assess biological activity. In this regard, studies in the DRG X neuroblastoma F-11 cell line revealed that while both AEA and 2-AG are present, they exhibit distinct subcellular distributions: AEA is found in both lipid raft and non-raft membrane fractions, whereas 2-AG is primarily localized within lipid rafts [74].

A key feature of endocannabinoids is their ability to be released on demand in response to local neuronal depolarization [64]. In this regard, the above-described basal levels of endocannabinoids are likely altered by cortical spreading depolarization (CSD). CSD is a wave of neuronal and glial depolarization followed by a period of electrical silence that is associated with the aura phase of migraine [75]. In an *ex vivo* rat CSD model, the depolarization wave is triggered in cortical slices by a high concentration of KCl. In this model, cortical AEA levels and release increased markedly, while 2-AG levels remained unchanged [76]. This nicely aligns with other findings showing that cultured neurons synthesize AEA upon depolarization [77]. The same group and Stella et al. reported that neurons also release 2-AG in a Ca^2+^-dependent manner [78], with brain concentrations reaching up to 170-fold higher than AEA [79]. Consistent with this higher 2-AG abundance, inhibition of serine hydrolases with AKU-005 leads to a more pronounced elevation in 2-AG levels compared to AEA in the rat cortex [76]. However, it is important to consider species-specificity of the ECS. In mouse cortical samples, basal concentrations of AEA and 2-AG are very low and remain unchanged following CSD [76]. Yet, as observed in rat cortex, 2-AG levels markedly increase upon serine hydrolase inhibition with AKU-005 [76]. In the CNS, astrocytes similarly produce 2-AG [78], AEA, and other acylethanolamides [80], with synthesis dependent on Ca^2+^ and enhanced by endothelin-1 [80,81] or ATP [82]. Microglia, via P2X7 receptors expressed only when activated, also respond to ATP, generating ∼20 times more endocannabinoids than neurons or astrocytes [80,83,84]. Tissue injury further promotes endocannabinoid and eicosanoid release [[85], [86], [87]].

Similar to the cortical response, in the meninges only AEA release is specifically enhanced by depolarizing high-potassium treatment [64]. The cell types underlying these levels were not identified in this study. While endocannabinoids are known to modulate immune cell activation [46], it remains unclear whether immune cells can release endocannabinoids into the meninges. Additionally, it has been shown that beyond glia and neurons, cerebrovascular endothelial cells can also release AEA when stimulated with the Ca^2+^ ionophore A23187 [88].

### Activity of FAAH and MAGL in migraine-relevant areas

The levels of the endocannabinoids are maintained at physiological levels by the activity of their main degrading enzymes FAAH and MAGL. These enzymes have distinct profiles in various migraine related areas. FAAH and MAGL are present in the trigeminovascular system but show differential activity (Fig. 1).

Within the trigeminovascular system, FAAH and MAGL have been measured in the trigeminal ganglia and meninges. In rat trigeminal ganglia, there is high MAGL activity, with the short MAGL isoform being more active than the long isoform [25]. The opposite pattern is observed in the meninges, where FAAH activity is robust and MAGL activity is not detectable across species, including rats, mice, and humans [64,89]. The brainstem overall exhibits relatively low activity levels of both enzymes [25]. In this study, the entire brainstem was collected rather than isolating the TNC specifically. However, it was elsewhere reported that mouse TNC has a high MAGL expression [90], whereas in the rat PAG, another brainstem nucleus, MAGL is only moderately expressed [91]. Additionally, although direct measurements are lacking, the effective inhibition of FAAH in the PAG implies that this nucleus also contains at least moderate levels of FAAH activity [92].

Beyond the trigeminovascular system, both MAGL and FAAH are abundant in the rodent cerebellum, cortex, amygdala, and hippocampus [25,76,[93], [94], [95]], while they are very low in the murine thalamus and hypothalamus [[93], [94], [95], [96]]. A pattern similar to the trigeminal ganglia is observed in the rat lumbar spinal cord, where MAGL activity is relatively high. In contrast, MAGL activity in the cervical and thoracic spinal cord is substantially lower and is only slightly higher than FAAH [25,89]. Finally, in the CNS, α/β-hydrolase domain-containing 6 (ABHD6), another 2-AG-degrading enzyme has been shown to be active in the rat cortex [76] and expressed in the PAG as well [91].

Overall, Fig. 1 illustrates the regions of the CNS and PNS in a example rodent brain where various components of the ECS are expressed and active. A rodent brain was selected because, although CB1 and CB2 receptor expression has been extensively characterized and is largely translatable to humans, much less is known about endocannabinoid levels and the activity of their degrading enzymes in human tissues. The detailed profile of the endocannabinoid components in migraine relevant areas mostly comes from rodent studies. Additionally, Fig. 1 is designed to parallel the figure published by Kuburas et al. which mapped the localization of the primary migraine triggers pituitary adenylate cyclase-activating polypeptide (PACAP) and CGRP along with their receptors in the human brain [97]. Our goal is to highlight the areas of the CNS and PNS where migraine-related signaling and the ECS overlap. This overlap is evident in most areas, with a notable exception being the sphenopalatine ganglia (SPG). While PACAP is highly expressed in the SPG [98,99], to date, whether ECS ligands or receptors are even present in the SPG has not been reported. Hence the ECS in the sphenopalatine ganglia is an untapped future area for study. We also aim to highlight key gaps in knowledge, the first being the limited awareness of species-specific differences and the very limited availability of human data. Moreover, the comparison between the two figures has the scope to show regions implicated in migraine where the presence of ECS elements has not yet been established, as well as areas rich in ECS components where their role in migraine remains unclear.

## Key preclinical studies targeting the endocannabinoid system for improving migraine-like symptoms

An increasing body of research supports the concept of Clinical Endocannabinoid Deficiency (CED). CED is the hypothesis that diminished endocannabinoid signaling and tone, particularly involving AEA and 2-AG, may underlie the mechanisms of functional pain disorders such as migraine [18,100]. Despite this, no therapeutic agents targeting CED have yet advanced to clinical trials. Notably, a recent Phase I clinical trial for the MAGL inhibitor ABX-1431 has been completed for testing its ability to treat patients affected by central pain disorders [101]. However, the trial results have not been posted yet and its potential clinical effects for migraine and headache still have to be considered. Nonetheless, preclinical research is ongoing (Table 1) and actively driving the field toward translational breakthroughs.

**Table 1:** Serine hydrolases inhibitors and their effect on migraine-like symptoms in rodent models.

	Inhibitors	Symptoms improved	Inj. route and dose	Time inj. from migraine trigger	Time inj. before effect	Sex differences
FAAH	URB597 [109]	NTG-induced hyperalgesia in orofacial formalin test	Acute 1 mg/kg, i.p.	Treating 3 h post- and preventing 1 h pre-NTG	Treating 1 h and preventing 5 h before rescue	Only male rats tested
	ARN14633 [110]	NTG-induced hyperalgesia in orofacial formalin test	Acute 1 mg/kg, i.p.	Treating 3 h post-NTG	Treating 1 h before rescue	Only male rats tested
	ARN14280 [110]		Acute 3 mg/kg, i.p.	Treating 3 h post-NTG	Treating 1 h before rescue	
	URB937 [111,112]	- Increase in NTG-induced tail flick latency - NTG-induced hyperalgesia in plantar and orofacial formalin test	Acute 1 mg/kg, i.p.	Treating 3 h post-NTG	Treating 1 h before rescue	Only male rats tested
		NTG-induced hyperalgesia in orofacial formalin test	- Acute 1 mg/kg, i.p. - Chronic 1 mg/kg, i.p.	- Acute: Treating 3 h post- and preventing 1 h pre-NTG - Chronic: concomitant NTG inj. every other day for 9 days	- Acute: Treating 1 h and preventing 5 h before rescue - Chronic: daily for 9 days	
	JZP327A [66]	NTG-induced hyperalgesia in orofacial formalin test	Acute 0.5 mg/kg, i.p.	Treating 3 h post-NTG	Treating 1 h before rescue	Only male rats tested
MAGL	URB602 [113]	- Increase in NTG-induced tail flick latency - NTG-induced hyperalgesia in plantar and orofacial formalin test	Acute 2 mg/kg, i.p.	Treating 3 h post-NTG	Treating 1 h before rescue	Only male rats tested
	JZL-184 [113]	NTG-induced hyperalgesia in orofacial formalin test	Acute 4 mg/kg, i.p.	Treating 3 h post-NTG	Treating 1 h before rescue	
	ABD-1970 [90]	Periorbital mechanical allodynia	- Acute 10 mg/kg, i.p. - Chronic 10 mg/kg, i.p.	- Acute: preventing 2 h pre-NTG - Chronic: on 1 acute NTG inj on the last day.	- Acute: preventing 4 h before rescue - Chronic: preventing daily for 5 days	No sex-differences (C57BL6/J mice)
	MJN110 [91]	Periorbital mechanical allodynia	Acute 10 mg/kg, cortical cannula injection in PAG	Treating 30min post- and preventing 30min pre-KCl	Treating 3 h and preventing 6 h 30min before rescue	Only female rats tested
ABHD6	KT-182 [91]	Periorbital mechanical allodynia	Acute 2 mg/kg, cortical cannula injection in PAG	Treating 30min post- and preventing 3 h pre-KCl	Treating 1 h and preventing 4 h before rescue	Only female rats tested
MAGL and FAAH	JZL195 [120]	NTG-induced hyperalgesia in orofacial formalin test	Acute 3 mg/kg, i.p.	Treating 2 h post-NTG	Treating 2 h before rescue	Only male rats tested
MAGL, FAAH, and ABHD6	AKU-005 [65,76]	NTG-induced hyperalgesia in orofacial formalin test	Acute 0.5 mg/kg, i.p.	Treating 3 h post-NTG	Treating 1 h before rescue	Only male rats tested

Strategies to modulate the endocannabinoid system include increasing endocannabinoid synthesis or reducing their degradation. Evidence shows that mRNA levels of NAPE-PLD and DAGL, key enzymes for AEA and 2-AG synthesis, are elevated in both episodic migraine and chronic migraine with medication overuse, suggesting a compensatory attempt to boost endocannabinoid production in response to reduced endocannabinoid tone observed in migraine patients [102]. However, these patients also show increased MAGL expression, indicating enhanced 2-AG breakdown that may offset any rise in synthesis [102]. Because this compensatory synthesis is likely counteracted by accelerated degradation, the main strategy to potentially treat migraine symptoms has been so far to target endocannabinoid hydrolases to increase endocannabinoids levels and deal with the CED.

The following potential therapeutic compounds have been tested in rodent migraine models in which migraine-like attacks were induced by nitroglycerin (NTG), a nitric oxide donor known to trigger migraine attacks in patients [103]. Another relevant model is the CSD model in rats and mice as previously described [104]. In migraine models, rodent migraine-like symptoms are evaluated using several established assays. The plantar and orofacial formalin tests assess hyperalgesia, a common feature of migraine that is exacerbated by prior NTG administration [105]. A caveat is that the formalin is a very noxious stimulus that produces several effects, including tissue injury. However, it is commonly used since it activates trigeminal and spinal Aδ and C fiber nociceptive neurons to produce a painful sensation in humans [106]. Facial sensitivity to von Frey filaments is generally used as a measure of periorbital mechanical allodynia, another common migraine symptom [107]. Future studies are needed to test these compounds across additional migraine models and symptoms.

### FAAH inhibitors

FAAH is the first discovered endocannabinoid serine hydrolase and it is responsible for the degradation of AEA and other N-acyl ethanolamines, such as palmitoylethanolamide (PEA) and oleoylethanolamide (OEA) [108]. Importantly, the FAAH inhibitor URB597 reduced NTG-induced orofacial hyperalgesia in rats [109]. At the molecular level, URB597 increased AEA and PEA levels in the medulla, cervical spinal cord, and TG. When administered as a pretreatment, it prevented the NTG- and formalin-induced upregulation of CGRP, nNOS, IL-1β, and TNF-α in both the medulla and spinal cord. However, URB597 was ineffective when given post-NTG, suggesting its effects are time-dependent [109]. The URB597 analogs ARN14633 and ARN14280, designed for better solubility and bioavailability, reduced NTG-induced nocifensive behavior and suppressed expression of nNOS, CGRP, substance P, TNF-α, IL-1β, and IL-6 in the trigeminal ganglion, cervical spinal cord, and medulla [110]. NTG alone did not alter FAAH substrate levels in these regions, but both compounds significantly increased AEA, PEA, and OEA levels in the cervical spinal cord and medulla, regardless of NTG pre-treatment [110]. Similarly, the peripherally restricted FAAH inhibition by URB937 reduced NTG-induced trigeminal hyperalgesia in a migraine model. This effect was observed when the compound was administered either acutely, 1 h before or 3 h after NTG injection, with testing performed 4 h post-NTG, or chronically, once daily for 9 days, in parallel with repeated NTG administration every other day [111,112]. Matching the behavioral outcome, URB937 increased anandamide levels in the trigeminal ganglia, particularly when given before NTG, but not in the medulla. Additionally, URB937 reduced expression of CGRP, TNF-α, and IL-6 mRNA in the trigeminal ganglia and medulla, with stronger effects when administered after NTG [112]. Finally, another FAAH inhibitor, JZP327A also significantly reduced hyperalgesia in the orofacial formalin test. This compound strongly suppressed CGRP, TNF-α, and IL-6 mRNA gene expression in the trigeminal ganglia and medulla-pons, although without altering endocannabinoid levels, nor CGRP serum concentrations [66]. This outcome leaves open the question of the underlying mechanism driving the reduction in nocifensive behavior. It may be independent of endocannabinoid signaling, or alternatively, related effects could have gone undetected due to suboptimal sensitivity at the specific time point, compound concentration, or brain regions analyzed.

Overall, FAAH inhibitors, such as URB597, URB937, ARN14633, ARN14280, and JZP327A, all consistently reduced hyperalgesia and downregulated pro-nociceptive and inflammatory mRNA gene expression in trigeminal ganglia, spinal cord, and medulla. However, these studies were all done by the same research group, using only one preclinical migraine model of NTG followed by formalin-induced hyperalgesia, and included only male rats. Thus, these findings offer a limited view that may restrict their broader translational relevance.

### MAGL and ABHD6 inhibitors

MAGL and ABHD6 are the main endocannabinoid serine hydrolases that degrade 2-AG [23]. The reversible MAGL inhibitor URB602 effectively reduced NTG-induced hyperalgesia in both the tail flick and formalin tests across hind paw and orofacial regions in rats [113]. These results were coupled with a reduction of NTG-induced c-Fos expression in the TNC and PAG. Similarly, the MAGL inhibitor JZL184 also reduced NTG-induced hyperalgesia in the orofacial formalin test. However, when administered alone, both URB602 and JZL184 unexpectedly enhanced pain sensitivity in the trigeminal region following formalin injection [113]. Additionally, the MAGL-inhibitor ABD-1970 effectively blocked cephalic allodynia triggered by both acute and chronic intermittent NTG administration similarly in male and female mice [90]. Its anti-allodynic effects were maintained over repeated dosing for five days, showing no signs of tolerance development [90].

Lastly, pharmacological inhibition of MAGL by MNJ110 or ABHD6 by KT-182 both prevented and reversed periorbital allodynia associated with CSD induction [91]. Furthermore, this study shows that MAGL inhibition prevents and reverses CSD-induced allodynia via a CB2-dependent mechanism, while ABHD6 blockade exerts a similar but more time-dependent effect on periorbital allodynia, which appears independent on CB1 and CB2 receptors [91].

Overall, MAGL and ABHD6 inhibitors have demonstrated consistent anti-allodynic and anti-hyperalgesic effects across diverse migraine models, including NTG and CSD, introducing new outcomes like periorbital allodynia and employing both peripheral and central routes of administration. Compared to FAAH, MAGL-targeting studies offer greater experimental variety, but testing the same compound across additional models and behavioral readouts would enhance translational relevance.

In contrast to the findings supporting MAGL as a potential target for headache analgesia, NTG-induced periorbital allodynia was not reduced in MAGL knock-out mice, unlike the attenuation observed in FAAH knock-out mice [114]. This raises questions about the exclusive role of MAGL in mediating analgesic effects and suggests that the efficacy of MAGL inhibitors may also involve additional, as yet unidentified, mechanisms. Importantly, MAGL knock-out mice not only display elevated 2-AG levels but also develop CB1 receptor desensitization together with anxiety- and depression-like phenotypes [115]. Such alterations in CB1 receptor signaling, combined with heightened anxiety, could significantly confound behavioral assays such as periorbital allodynia. Additionally, consistent with this, repeated administration of the MAGL inhibitor JZL184 also induced CB1 receptor desensitization, resulting in a loss of analgesic activity and cross-tolerance to CB1 agonists, thereby mirroring the phenotype of genetic Mgll disruption, encoding for MAGL [116]. This appears to be very different from the chronic pharmacological inhibition of FAAH, which caused sustained analgesic effects without tolerance or changes in CB1 receptor expression or function [116]. This divergence may relate to 2-AG acting as a full CB1 receptor agonist, while anandamide functioning only as a partial agonist [117]. However, the precise mechanisms underlying these distinct adaptive responses remain unclear, as THC is likewise only a partial CB1 receptor agonist and yet still produces tolerance [118].

### Multi-hydrolase inhibitors

More recently, the distinct regional distribution of endocannabinoids and their degrading enzymes across areas relevant for migraine pathways (as discussed in section Endocannabinoid system in migraine pathways) has increased the interest in the development and testing of inhibitors targeting multiple enzymes simultaneously, aiming to achieve broader endocannabinoid enhancement and therapeutic effects.

A promising future therapeutic is the triple MAGL/FAAH/ABHD6 inhibitor AKU-005. AKU-005 was initially classified as a dual MAGL/FAAH inhibitor [64,65,119], but was later found able to also target ABHD6 [76]. AKU-005 reduced NTG-induced hyperalgesia in the orofacial formalin test [65]. AKU-005 also suppressed serum CGRP levels and pro-inflammatory cytokine mRNA expression in the meninges, TG, cervical spinal cord, and medulla through an unknown mechanism. Unexpectedly, it did not alter endocannabinoid levels in any of these regions [65]. Similarly to the FAAH-inhibitor JZP327A, AKU-005 may act independently of endocannabinoid signaling or have effects not captured due to timing, dose, or region-specific limitations. AKU-005 has been shown to increase AEA and 2-AG levels in *ex vivo* cortical slices from both mice and rats [76]. However, the cortex was not examined in the *in vivo* study described above. It is also possible that limited brain penetration or rapid metabolism of the compound *in vivo* may have prevented detectable endocannabinoid elevation.

Along the same line as AKU-005, the dual MAGL/FAAH-inhibitor JZL195 reduced NTG-induced trigeminal orofacial hyperalgesia in the formalin test [120]. This behavioral effect was accompanied by reduced expression of CGRP and pro-inflammatory cytokines in TG and cervical spinal cord, as well as lower circulating CGRP levels in the plasma [120]. However, endocannabinoid levels were not assessed following JZL195 treatment.

Overall, the multi-hyrdolase enzyme inhibitors AKU-005 and JZL195 reduced NTG-induced orofacial hyperalgesia and inflammation-related markers, supporting their potential in migraine models. However, endocannabinoid levels were not measured with JZL195 and not detected with AKU-005. Finally, all studies *in vivo* were conducted by the same research group using exclusively the NTG migraine model in male rats, limiting broader translational relevance.

## Molecular targets in the endocannabinoid system for migraine treatment

### CB1 and CB2 receptors

Targeting CB1 and CB2 receptors with selective agonists or antagonists represents an alternative strategy for developing new migraine treatments. This connection is illustrated by data showing that the CB1 inverse agonist AM251 reversed both AEA and 2-AG inhibition of KCl-induced firing of meningeal nerve afferents in rats [64] and mice [89]. Firing was unaffected by the TRPV1 antagonist capsazepine. AEA has also been shown to inhibit trigeminocervical complex neuron activity via CB1, while simultaneously acting as a TRPV1 agonist that can facilitate CGRP release [[121], [122], [123]]. These findings suggest that selectively modulating CB1 without engaging TRPV1 could offer therapeutic benefit.

Cannabinoid receptor activity may also be modulated by minor endocannabinoids such as PEA and OEA. Indeed, PEA levels have been reported to be significantly higher in individuals with chronic migraine than in healthy controls, suggesting that this increase may represent a compensatory response to reduced endocannabinoid system tone in chronic migraine [124]. Several FAAH inhibitors listed in Table 1 are known to raise not only AEA but also these smaller lipid mediators. This suggests that future therapeutic approaches could benefit from targeting these minor endocannabinoid lipids as well.

More recently, allosteric endocannabinoids such as pregnenolone and lipoxin A4 have been identified as CB1 receptor signaling modulators, suggesting potential therapeutic value [[125], [126], [127]]. This mirrors the combined effectiveness of THC, which binds CB1/2 receptors orthosterically, and CBD, which acts instead allosterically, neither of which alone, but only together, successfully reversed non-evoked pain and photophobia in CGRP-induced migraine mouse models [12]. Further pharmacological characterization is still needed of orthosteric and allosteric modulators to clearly elucidate their physiological roles and modes of action. Nevertheless, the pharmacological manipulation of endocannabinoid levels or their actions by allosteric modulators could provide alternative opportunities to regulate the analgesic role of the endocannabinoid signaling.

CB2 receptor activation has been shown to reduce nociceptive responses in various preclinical pain models without inducing tolerance [128,129] or causing central nervous system side effects [129], positioning CB2 agonists as promising candidates for pain therapy. In the context of migraine models, the selective CB2 agonist AM1241 produced significant antinociceptive effects under baseline conditions in both the tail flick and formalin tests [130]. Notably, when administered after NTG, AM1241 significantly reduced hyperalgesic behaviors [130].

### Targets beyond CB1/2

Endocannabinoid signaling offers novel receptor targets for potential migraine treatment beyond classical CB1 and CB2 activation. Stimulation by dopaminergic input to the TNC may enhance CGRP release indirectly through AEA mobilization and subsequent TRPV1 activation, forming a dopamine–AEA–CGRP axis [131]. Dopamine-induced CGRP release was significantly reduced either by blocking TRPV1 or by inhibiting the AEA-synthetizing enzyme N-acyl phosphatidylethanolamine phospholipase D (NAPE-PLD) [131]. These findings also expand the therapeutic landscape to include dopamine D2 receptors, which have been shown to regulate AEA release in other brain regions [132,133]. Furthermore, activation of CB1 and CB2 receptors in the ventrolateral periaqueductal gray (vlPAG) using WIN55,212 and the CB1-selective agonist ACPA significantly reduced dural Aδ-fiber activity in the rat trigeminocervical complex, without altering facial cutaneous receptive field responses [26]. These inhibitory effects were reversed by intra-vlPAG administration of the CB1 antagonist SR141716, and by the 5-HT1B/1D receptor antagonist GR127935 [26]. Together, these results point to a functional interplay between the endocannabinoid and serotonergic systems in regulating somatosensory nociceptive input and suggest a new therapeutical targets of endocannabinoid-signaling neurons within the vlPAG. Moreover, CB1 receptors are well known to modulate inwardly rectifying potassium channels, and endocannabinoids have been shown to influence a variety of potassium channel subtypes, as extensively reviewed [134]. In trigeminal neurons, AEA was shown to reduce GABA-A receptor activity, which normally exerts inhibitory control via chloride channels [135]. Additionally, endocannabinoids may act as CBD, which inhibits Nav channels [136,137], effect that may dampen action potential propagation in nociceptive pathways. Whether AEA or 2-AG exert comparable effects on sodium channels in any region relevant to migraine signaling remains to be explored. These studies highlight the potential to target specific ion channels influenced by endocannabinoids as an alternative approach to modulate neuronal excitability in migraine pathways.

**Fig. 2:**
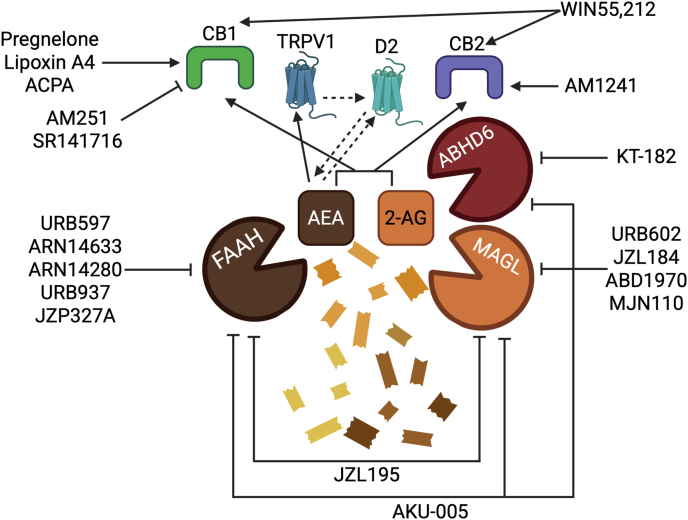
**Activators and inhibitors of the endocannabinoid system components.** Activator and inhibitor compounds described in sections Key preclinical studies targeting the endocannabinoid system for improving migraine-like symptoms and Molecular targets in the endocannabinoid system for migraine treatment, Key preclinical studies targeting the endocannabinoid system for improving migraine-like symptoms and Molecular targets in the endocannabinoid system for migraine treatment, that have been tested for cannabinoid receptor type 1/2 (CB1/CB2) modulation or inhibition of the endocannabinoid-degrading enzymes fatty acid amide hydrolase (FAAH), monoacylglycerol lipase (MAGL), and α/β-hydrolase domain containing 6 (ABHD6) are shown in the context of potential future development of migraine analgesic drugs. Only two compounds, Pregnelone and Lipoxin A4, have been shown to act as allosteric modulators of CB1 receptor. Within the endocannabinoid system, anandamide (AEA) and 2-arachidonoylglycerol (2-AG) activate CB1 and CB2, and are degraded by FAAH and ABHD6 and MAGL, as indicated. At high concentrations, AEA can also stimulate transient receptor potential vanilloid type 1 (TRPV1). TRPV1 receptor activation and AEA may be linked to modulation of dopamine D2 receptor (D2) signaling, as their blockage reduces dopamine-induced calcitonin gene-related peptide (CGRP) release. D2 receptors can regulate AEA release in some brain regions.

To summarize, beside inhibiting endocannabinoid degrading enzymes, targeting cannabinoid receptors and endocannabinoid-modulated ion channels offers promising new strategies for migraine treatment (Fig. 2). Emerging evidence supports roles for CB1, CB2, TRPV1, dopamine D2 receptors, and downstream signaling pathways in modulating trigeminovascular activity, CGRP release, and neuronal excitability.

## Endocannabinoids and sleep-related circadian and glymphatic rhythms

The ECS is involved in numerous interconnected homeostatic functions that may be relevant to migraine. Two sleep-related areas in which endocannabinoids may represent a promising therapeutic avenue are modulation of circadian regulation [138] and glymphatic clearance [57]. These are two interlinked systems that are emerging as recognized contributors to migraine pathophysiology. In addition, the ECS is a major player in systemic energy metabolism, inflammation, appetite control, and pleasure during exercise [139]. For example, diet affects plasma endocannabinoid levels [140]. Accordingly, lifestyle behaviors such as maintaining regular sleep, following a balanced diet, and engaging in consistent physical activity have been shown to help reduce migraine frequency and severity [141].

### Endocannabinoids resetting the clock in migraine

**Fig. 3:**
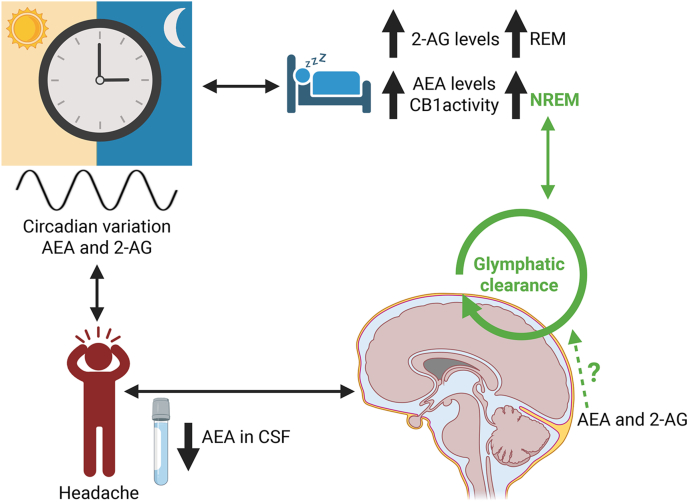
**Endocannabinoid system potential effect on the link between circadian regulation, sleep, glymphatic clearance, and headache.** The endocannabinoids AEA and 2-AG may contribute to migraine pathophysiology through their dual role in circadian and glymphatic regulation. In healthy conditions, AEA and 2-AG display circadian oscillations that influence hypothalamic circuits and CB1 receptor activity. 2-AG levels increase during REM sleep. Increased CB1 activity promotes NREM sleep, when the highest AEA levels have been observed and during which glymphatic clearance peaks. In migraine, cerebrospinal fluid (CSF) levels of AEA are reduced, which may impair sleep quality and disrupt clearance mechanisms, perpetuating headache symptoms. Whether endocannabinoids directly modulate glymphatic flow remains unresolved (green dashed line with question mark).

The ECS, particularly through CB1 receptor signaling, is known to influence hypothalamic circuits involved in circadian control [142,143]. In healthy individuals, circulating and tissue levels of the endocannabinoids AEA and 2-AG display circadian variation of their own with area-specific variations [[143], [144], [145]] (Fig. 3). The highest AEA levels have been observed during the non-rapid eye movement (NREM) sleep, while 2-AG levels increase during rapid eye movement (REM) sleep [146,147]. Dysregulation of these systems is commonly reported in migraine patients, many of whom exhibit altered sleep patterns and chronobiological disturbances [148,149]. The ECS influences sleep also through the activity of CB1 receptors. The pharmacological activation of CB1 receptors has been shown to enhance NREM, when glymphatic flow activity is at its highest [150], by prolonging and stabilizing individual sleep bouts. Conversely, inhibition of CB1 receptors with the antagonist AM251 leads to fragmentation of NREM [151] (Fig. 3). Moreover, the activation of CB1 receptors promotes GABAergic transmission, which supports both the initiation of sleep and the stability of non-REM phases. Additionally, CB1 regulation of glutamate release influences wakefulness, highlighting the ECS's dual function in maintaining the balance of the sleep-wake cycle [152,153]. Finally, also inhibiting the endocannabinoid degrading enzymes MAGL and FAAH may help modulating sleep. For instance, the increased endocannabinoids levels resulting from this inhibition has been shown to facilitate sleep onset and improve its continuity [[154], [155], [156]].

### Possible endocannabinoid roles in glymphatic clearing of the brain in migraine

The ECS may help regulate the glymphatic clearance pathway. The ECS appears to play at least an indirect role in regulating glymphatic flow, a sleep-dependent system implicated in migraine. The glymphatic system is a brain-wide clearance mechanism of cerebrospinal fluid (CSF) that operates most efficiently during sleep to eliminate metabolic waste [[157], [158], [159]]. Specifically, CSF movement in the glymphatic pathway occurs along the perivascular space, between the brain vasculature and astrocytic end-feet [160,161]. Notably, AEA levels are reduced in the CSF of individuals with chronic migraine [124] (Fig. 3), suggesting that impaired endocannabinoid tone may contribute to migraine persistence. Restoring physiological CSF endocannabinoid levels could therefore offer a therapeutic strategy. However, other than the presence of endocannabinoids in the CSF, a direct connection between the ESC and glymphatic flow remains uninvestigated.

An indirect connection between the ECS and glymphatic flow is likely since the ECS has been linked to blood-brain barrier (BBB) modulation and the glymphatic system is closely associated with the astrocytes, pericytes and endothelial cells that constitute the BBB [160,161]. BBB integrity is essential for driving water movement via astrocytic aquaporin 4 (AQP4) channels from intra- and extracellular brain compartments into the vasculature [162]. This water flow drives the perivascular CSF movement in the glymphatic system. Astrocytes express CB1 and CB2 receptors [163], although the physiological relevance of CB2 remains debated [60], and the MAGL and FAAH enzymes that degrade AEA and 2-AG [164,165]. The astrocytic control of endocannabinoid levels has implications for neural circuitry, synaptic plasticity, and overall brain homeostasis [[166], [167], [168]]. In mice models, activating CB2 receptor suppressed inflammation caused by TBI, prevented BBB damage [169,170]. The CB2 selective agonist JWH133 could protect the BBB integrity by increasing the expression of the intercellular adhesion molecule 1 [171]. Thus, ECS modulation of BBB permeability and hence glymphatic flow could represent a promising therapeutic avenue for cannabinoid-based interventions in migraine and migraine-like headache following brain injury. A glymphatic impairment has been proposed in post-traumatic headache (PTH), a migraine-like headache [172]. An increased level of 2-AG was observed in TBI mouse models [173]. Additionally, enhancing 2-AG signaling following TBI has demonstrated the ability to reduce neuroinflammation and brain edema [174,175], which could facilitate glymphatic restoration and support their use in headache disorders with disrupted waste clearance. Whether the same mechanism for endocannabinoids to restore glymphatic flow could help with migraine symptoms has so far not been investigated. However, we could hypothesize that, given the ECS dual regulatory role in circadian timing and glymphatic function, pharmacological modulation of endocannabinoid signaling may “reset the clock” and “clear the brain” in migraine, offering a compelling new strategy.

Overall, given that glymphatic and sleep impairment has been implicated in both post-traumatic headache and migraine, and that endocannabinoids can modulate both these pathways, targeting the ECS presents a promising opportunity to alleviate migraine by simultaneously restoring circadian–sleep regulation and enhancing glymphatic function.

## Conclusions and future perspectives

Current evidence shows that the ECS is embedded in multiple migraine-relevant pathways areas across the CNS and PNS. Preclinical studies demonstrate that enhancing endocannabinoid tone via inhibition of endocannabinoid-degrading hydrolases reduces migraine-like pain and neuroinflammation. Beyond these analgesic strategies, targeting cannabinoid receptors, modulating their activity, and influencing their modulation of circadian regulation and glymphatic clearance further highlight the therapeutic potential of engaging the ECS.

Based on the current literature evidence and gaps in knowledge, future research should focus on multi-target inhibitors to achieve broader and region-specific endocannabinoid enhancement. To strengthen translational value, candidate compounds should be tested across different strains, in both sexes, and in diverse migraine models, with expanded behavioral and physiological assessments.

## Author contributions

Conceptualization, ADP and AFR; writing—original draft preparation, ADP.; writing—review and editing, ADP and AFR; figures design, ADP.; supervision, AFR. All authors have read and agreed to the published version of the manuscript.

## Declaration of competing interest

The authors declare the following financial interests/personal relationships which may be considered as potential competing interests: Andrew F. Russo reports financial support was provided by National Institutes of Health R01NS129573. Adriana Della Pietra reports financial support was provided by HORIZON-MSCA-2024-PF-01 101208555. Adriana Della Pietra reports a relationship with Delphian therapeutics that includes: consulting or advisory. Andrew F. Russo reports a relationship with Lundbeck that includes: consulting or advisory. Andrew F. Russo reports a relationship with Abbvie that includes: consulting or advisory. Andrew F. Russo reports a relationship with Pfizer that includes: consulting or advisory. Andrew F. Russo reports a relationship with Vedana Therapeutics that includes: consulting or advisory. Andrew F. Russo reports a relationship with Arrowhead Pharmaceuticals that includes: consulting or advisory. Andrew F. Russo reports a relationship with Omeros Corporation that includes: consulting or advisory. Andrew F. Russo reports a relationship with Solros Therapeutics that includes: consulting or advisory. Andrew F. Russo reports a relationship with Delphian Therapeutics that includes: consulting or advisory. If there are other authors, they declare that they have no known competing financial interests or personal relationships that could have appeared to influence the work reported in this paper.

## References

[1] Steiner T.J., Stovner L.J. (2023). Global epidemiology of migraine and its implications for public health and health policy. Nat Rev Neurol.

[2] Leonardi M., Raggi A. (2019). A narrative review on the burden of migraine: when the burden is the impact on people's life. J Headache Pain.

[3] Gil-Gouveia R. (2021). “Headache yesterday” at work. Pilot study of headache impact in an active workforce. Rev Neurol (Paris).

[4] Steiner T.J., Stovner L.J., Jensen R., Uluduz D., Katsarava Z. (2020). Lifting the burden: the Global Campaign against H. Migraine remains second among the world's causes of disability, and first among young women: findings from GBD2019. J Headache Pain.

[5] Steiner T.J., Stovner L.J., Vos T., Jensen R., Katsarava Z. (2018). Migraine is first cause of disability in under 50s: will health politicians now take notice?. J Headache Pain.

[6] Hong J.B., Lange K.S., Overeem L.H., Triller P., Raffaelli B., Reuter U. (2023). A scoping review and meta-analysis of Anti-CGRP monoclonal antibodies: predicting response. Pharmaceuticals (Basel).

[7] Lucas C.J., Galettis P., Schneider J. (2018). The pharmacokinetics and the pharmacodynamics of cannabinoids. Br J Clin Pharmacol.

[8] Zhang N., Woldeamanuel Y.W. (2021). Medication overuse headache in patients with chronic migraine using cannabis: a case-referent study. Headache.

[9] Rhyne D.N., Anderson S.L., Gedde M., Borgelt L.M. (2016). Effects of medical Marijuana on Migraine Headache frequency in an adult population. Pharmacotherapy.

[10] Crocq M.A. (2020). History of cannabis and the endocannabinoid system. Dialogues Clin Neurosci.

[11] Atakan Z. (2012). Cannabis, a complex plant: different compounds and different effects on individuals. Ther Adv Psychopharmacol.

[12] Zorrilla E., Krivoshein G., Kuburas A., Schenke M., Pina C.L., van Heiningen S.H. (2025). Combined effects of cannabidiol and Delta9-tetrahydrocannabinol alleviate migraine-like symptoms in mice. Cephalalgia.

[13] Zorrilla E., Duong T.L., Pina C.L., Russo A.F. (2025). Cannabinoids rescue migraine symptoms caused by central CGRP administration in mice. Cephalalgia.

[14] Efficacy of inhaled cannabis for acute migraine treatment. https://clinicaltrials.gov/study/NCT04360044?term=Nathaniel%20Schuster&amp;rank=2.

[15] Schuster N.M., Wallace M.S., Marcotte T.D., Buse D.C., Lee E., Liu L. (2024). Vaporized Cannabis versus placebo for acute migraine: a randomized controlled trial. medRxiv.

[16] Matsuda L.A., Lolait S.J., Brownstein M.J., Young A.C., Bonner T.I. (1990). Structure of a cannabinoid receptor and functional expression of the cloned cDNA. Nature.

[17] Shao Z., Yan W., Chapman K., Ramesh K., Ferrell A.J., Yin J. (2019). Structure of an allosteric modulator bound to the CB1 cannabinoid receptor. Nat Chem Biol.

[18] Leimuranta P., Khiroug L., Giniatullin R. (2018). Emerging role of (Endo)Cannabinoids in migraine. Front Pharmacol.

[19] Bisogno T., Howell F., Williams G., Minassi A., Cascio M.G., Ligresti A. (2003). Cloning of the first sn1-DAG lipases points to the spatial and temporal regulation of endocannabinoid signaling in the brain. J Cell Biol.

[20] Pertwee R.G., Howlett A.C., Abood M.E., Alexander S.P., Di Marzo V., Elphick M.R. (2010). International Union of Basic and Clinical Pharmacology. LXXIX. Cannabinoid receptors and their ligands: beyond CB(1) and CB(2). Pharmacol Rev.

[21] Jin X.H., Okamoto Y., Morishita J., Tsuboi K., Tonai T., Ueda N. (2007). Discovery and characterization of a Ca2+-independent phosphatidylethanolamine N-acyltransferase generating the anandamide precursor and its congeners. J Biol Chem.

[22] Savinainen J.R., Saario S.M., Laitinen J.T. (2012). The serine hydrolases MAGL, ABHD6 and ABHD12 as guardians of 2-arachidonoylglycerol signalling through cannabinoid receptors. Acta Physiol (Oxf).

[23] Blankman J.L., Simon G.M., Cravatt B.F. (2007). A comprehensive profile of brain enzymes that hydrolyze the endocannabinoid 2-arachidonoylglycerol. Chem Biol.

[24] van Egmond N., Straub V.M., van der Stelt M. (2021). Targeting endocannabinoid signaling: FAAH and MAG lipase inhibitors. Annu Rev Pharmacol Toxicol.

[25] Della Pietra A., Giniatullin R., Savinainen J.R. (2021). Distinct activity of endocannabinoid-hydrolyzing enzymes MAGL and FAAH in key regions of peripheral and central nervous system implicated in migraine. Int J Mol Sci.

[26] Akerman S., Holland P.R., Lasalandra M.P., Goadsby P.J. (2013). Endocannabinoids in the brainstem modulate dural trigeminovascular nociceptive traffic via CB1 and "triptan" receptors: implications in migraine. J Neurosci.

[27] Moldrich G., Wenger T. (2000). Localization of the CB1 cannabinoid receptor in the rat brain. An immunohistochemical study. Peptides.

[28] Stella N. (2010). Cannabinoid and cannabinoid-like receptors in microglia, astrocytes, and astrocytomas. Glia.

[29] Straiker A., Mackie K. (2005). Depolarization-induced suppression of excitation in murine autaptic hippocampal neurones. J Physiol.

[30] Wu D.F., Yang L.Q., Goschke A., Stumm R., Brandenburg L.O., Liang Y.J. (2008). Role of receptor internalization in the agonist-induced desensitization of cannabinoid type 1 receptors. J Neurochem.

[31] Price T.J., Helesic G., Parghi D., Hargreaves K.M., Flores C.M. (2003). The neuronal distribution of cannabinoid receptor type 1 in the trigeminal ganglion of the rat. Neuroscience.

[32] Bridges D., Rice A.S., Egertova M., Elphick M.R., Winter J., Michael G.J. (2003). Localisation of cannabinoid receptor 1 in rat dorsal root ganglion using in situ hybridisation and immunohistochemistry. Neuroscience.

[33] Ford Z.K., Reker A.N., Chen S., Kadakia F., Bunk A., Davidson S. (2021). Cannabinoid receptor 1 expression in Human Dorsal root Ganglia and CB13-Induced bidirectional modulation of sensory neuron activity. Front Pain Res (Lausanne).

[34] Veress G., Meszar Z., Muszil D., Avelino A., Matesz K., Mackie K. (2013). Characterisation of cannabinoid 1 receptor expression in the perikarya, and peripheral and spinal processes of primary sensory neurons. Brain Struct Funct.

[35] Glass M., Dragunow M., Faull R.L. (1997). Cannabinoid receptors in the human brain: a detailed anatomical and quantitative autoradiographic study in the fetal, neonatal and adult human brain. Neuroscience.

[36] Zou S., Kumar U. (2018). Cannabinoid receptors and the endocannabinoid system: signaling and function in the central nervous system. Int J Mol Sci.

[37] Mackie K. (2005). Distribution of cannabinoid receptors in the central and peripheral nervous system. Handb Exp Pharmacol.

[38] Dai W., Liu R.H., Qiu E., Liu Y., Chen Z., Chen X. (2021). Cortical mechanisms in migraine. Mol Pain.

[39] Kosuge S., Masaoka Y., Kasai H., Honma M., Murakami K., Yoshii N. (2024). The right amygdala and migraine: analyzing volume reduction and its relationship with symptom severity. PLoS One.

[40] Maleki N., Becerra L., Brawn J., McEwen B., Burstein R., Borsook D. (2013). Common hippocampal structural and functional changes in migraine. Brain Struct Funct.

[41] Younis S., Hougaard A., Noseda R., Ashina M. (2019). Current understanding of thalamic structure and function in migraine. Cephalalgia.

[42] Kagan R., Kainz V., Burstein R., Noseda R. (2013). Hypothalamic and basal ganglia projections to the posterior thalamus: possible role in modulation of migraine headache and photophobia. Neuroscience.

[43] Wang M., Duong T.L., Rea B.J., Waite J.S., Huebner M.W., Flinn H.C. (2022). CGRP administration into the cerebellum evokes light aversion, tactile hypersensitivity, and nociceptive squint in mice. Front Pain Res (Lausanne).

[44] Berger A.A., Liu Y., Possoit H., Rogers A.C., Moore W., Gress K. (2021). Dorsal root ganglion (DRG) and chronic pain. Anesthesiol Pain Med.

[45] Munro S., Thomas K.L., Abu-Shaar M. (1993). Molecular characterization of a peripheral receptor for cannabinoids. Nature.

[46] Zorrilla E., Della Pietra A., Russo A.F. (2024). Interplay between cannabinoids and the neuroimmune system in migraine. J Headache Pain.

[47] Golech S.A., McCarron R.M., Chen Y., Bembry J., Lenz F., Mechoulam R. (2004). Human brain endothelium: coexpression and function of vanilloid and endocannabinoid receptors. Brain Res Mol Brain Res.

[48] Lu T.S., Avraham H.K., Seng S., Tachado S.D., Koziel H., Makriyannis A. (2008). Cannabinoids inhibit HIV-1 Gp120-mediated insults in brain microvascular endothelial cells. J Immunol.

[49] Gong J.P., Onaivi E.S., Ishiguro H., Liu Q.R., Tagliaferro P.A., Brusco A. (2006). Cannabinoid CB2 receptors: immunohistochemical localization in rat brain. Brain Res.

[50] den Boon F.S., Chameau P., Schaafsma-Zhao Q., van Aken W., Bari M., Oddi S. (2012). Excitability of prefrontal cortical pyramidal neurons is modulated by activation of intracellular type-2 cannabinoid receptors. Proc Natl Acad Sci USA.

[51] Xi Z.X., Peng X.Q., Li X., Song R., Zhang H.Y., Liu Q.R. (2011). Brain cannabinoid CB(2) receptors modulate cocaine's actions in mice. Nat Neurosci.

[52] Stempel A.V., Stumpf A., Zhang H.Y., Ozdogan T., Pannasch U., Theis A.K. (2016). Cannabinoid type 2 receptors mediate a cell type-specific plasticity in the hippocampus. Neuron.

[53] Palazuelos J., Aguado T., Pazos M.R., Julien B., Carrasco C., Resel E. (2009). Microglial CB2 cannabinoid receptors are neuroprotective in Huntington's disease excitotoxicity. Brain.

[54] Fernandez-Ruiz J., Moreno-Martet M., Rodriguez-Cueto C., Palomo-Garo C., Gomez-Canas M., Valdeolivas S. (2011). Prospects for cannabinoid therapies in basal ganglia disorders. Br J Pharmacol.

[55] Afridi R., Bhusal A., Tsuda M., Ryu H., Suk K. (2023). Function of glial cells in neuroinflammatory and neuroimmunological responses II. Cells.

[56] Donnelly C.R., Andriessen A.S., Chen G., Wang K., Jiang C., Maixner W. (2020). Central nervous system targets: glial cell mechanisms in chronic pain. Neurotherapeutics.

[57] Vittorini M.G., Sahin A., Trojan A., Yusifli S., Alashvili T., Bonifacio G.V. (2024). The glymphatic system in migraine and other headaches. J Headache Pain.

[58] Kofalvi A., Lemos C., Martin-Moreno A.M., Pinheiro B.S., Garcia-Garcia L., Pozo M.A. (2016). Stimulation of brain glucose uptake by cannabinoid CB2 receptors and its therapeutic potential in Alzheimer's disease. Neuropharmacology.

[59] Molina-Holgado F., Molina-Holgado E., Guaza C., Rothwell N.J. (2002). Role of CB1 and CB2 receptors in the inhibitory effects of cannabinoids on lipopolysaccharide-induced nitric oxide release in astrocyte cultures. J Neurosci Res.

[60] Lange S.C., Bak L.K., Waagepetersen H.S., Schousboe A., Norenberg M.D. (2012). Primary cultures of astrocytes: their value in understanding astrocytes in health and disease. Neurochem Res.

[61] Dowie M.J., Grimsey N.L., Hoffman T., Faull R.L., Glass M. (2014). Cannabinoid receptor CB2 is expressed on vascular cells, but not astroglial cells in the post-mortem human Huntington's disease brain. J Chem Neuroanat.

[62] Nunez E., Benito C., Tolon R.M., Hillard C.J., Griffin W.S., Romero J. (2008). Glial expression of cannabinoid CB(2) receptors and fatty acid amide hydrolase are beta amyloid-linked events in Down's syndrome. Neuroscience.

[63] Dhopeshwarkar A., Mackie K. (2014). CB2 Cannabinoid receptors as a therapeutic target-what does the future hold?. Mol Pharmacol.

[64] Della Pietra A., Krivoshein G., Ivanov K., Giniatullina R., Jyrkkanen H.K., Leinonen V. (2023). Potent dual MAGL/FAAH inhibitor AKU-005 engages endocannabinoids to diminish meningeal nociception implicated in migraine pain. J Headache Pain.

[65] Greco R., Demartini C., Francavilla M., Zanaboni A.M., Facchetti S., Palmisani M. (2024). Effects of the dual FAAH/MAGL inhibitor AKU-005 on trigeminal hyperalgesia in Male rats. Cells.

[66] Greco R., Francavilla M., Demartini C., Zanaboni A.M., Facchetti S., Palmisani M. (2023). Activity of FAAH-inhibitor JZP327A in an experimental rat model of migraine. Int J Mol Sci.

[67] Mitrirattanakul S., Ramakul N., Guerrero A.V., Matsuka Y., Ono T., Iwase H. (2006). Site-specific increases in peripheral cannabinoid receptors and their endogenous ligands in a model of neuropathic pain. Pain.

[68] Petrosino S., Palazzo E., de Novellis V., Bisogno T., Rossi F., Maione S. (2007). Changes in spinal and supraspinal endocannabinoid levels in neuropathic rats. Neuropharmacology.

[69] Kirkham T.C., Williams C.M., Fezza F., Di Marzo V. (2002). Endocannabinoid levels in rat limbic forebrain and hypothalamus in relation to fasting, feeding and satiation: stimulation of eating by 2-arachidonoyl glycerol. Br J Pharmacol.

[70] Jhaveri M.D., Elmes S.J., Richardson D., Barrett D.A., Kendall D.A., Mason R. (2008). Evidence for a novel functional role of cannabinoid CB(2) receptors in the thalamus of neuropathic rats. Eur J Neurosci.

[71] Hill M.N., McLaughlin R.J., Morrish A.C., Viau V., Floresco S.B., Hillard C.J. (2009). Suppression of amygdalar endocannabinoid signaling by stress contributes to activation of the hypothalamic-pituitary-adrenal axis. Neuropsychopharmacology.

[72] La Porta C., Bura S.A., Llorente-Onaindia J., Pastor A., Navarrete F., Garcia-Gutierrez M.S. (2015). Role of the endocannabinoid system in the emotional manifestations of osteoarthritis pain. Pain.

[73] Henry R.J., Kerr D.M., Finn D.P., Roche M. (2014). FAAH-mediated modulation of TLR3-induced neuroinflammation in the rat hippocampus. J Neuroimmunol.

[74] Rimmerman N., Hughes H.V., Bradshaw H.B., Pazos M.X., Mackie K., Prieto A.L. (2008). Compartmentalization of endocannabinoids into lipid rafts in a dorsal root ganglion cell line. Br J Pharmacol.

[75] Charles A.C., Baca S.M. (2013). Cortical spreading depression and migraine. Nat Rev Neurol.

[76] Brugia F., Ivanov K., Aroviita A., Giniatullina R., Lehtonen M., Malm T. (2025). Inhibition of endocannabinoid hydrolases MAGL, FAAH and ABHD6 by AKU-005 reduces ex vivo cortical spreading depression. J Headache Pain.

[77] Di Marzo V., Fontana A., Cadas H., Schinelli S., Cimino G., Schwartz J.C. (1994). Formation and inactivation of endogenous cannabinoid anandamide in central neurons. Nature.

[78] Stella N., Schweitzer P., Piomelli D. (1997). A second endogenous cannabinoid that modulates long-term potentiation. Nature.

[79] Bisogno T., Sepe N., Melck D., Maurelli S., De Petrocellis L., Di Marzo V. (1997). Biosynthesis, release and degradation of the novel endogenous cannabimimetic metabolite 2-arachidonoylglycerol in mouse neuroblastoma cells. Biochem J.

[80] Walter L., Franklin A., Witting A., Moller T., Stella N. (2002). Astrocytes in culture produce anandamide and other acylethanolamides. J Biol Chem.

[81] Walter L., Stella N. (2003). Endothelin-1 increases 2-arachidonoyl glycerol (2-AG) production in astrocytes. Glia.

[82] Walter L., Dinh T., Stella N. (2004). ATP induces a rapid and pronounced increase in 2-arachidonoylglycerol production by astrocytes, a response limited by monoacylglycerol lipase. J Neurosci.

[83] Walter L., Franklin A., Witting A., Wade C., Xie Y., Kunos G. (2003). Nonpsychotropic cannabinoid receptors regulate microglial cell migration. J Neurosci.

[84] Witting A., Walter L., Wacker J., Moller T., Stella N. (2004). P2X7 receptors control 2-arachidonoylglycerol production by microglial cells. Proc Natl Acad Sci U S A.

[85] Batkai S., Osei-Hyiaman D., Pan H., El-Assal O., Rajesh M., Mukhopadhyay P. (2007). Cannabinoid-2 receptor mediates protection against hepatic ischemia/reperfusion injury. FASEB J.

[86] Cao Z., Mulvihill M.M., Mukhopadhyay P., Xu H., Erdelyi K., Hao E. (2013). Monoacylglycerol lipase controls endocannabinoid and eicosanoid signaling and hepatic injury in mice. Gastroenterology.

[87] Nomura D.K., Morrison B.E., Blankman J.L., Long J.Z., Kinsey S.G., Marcondes M.C. (2011). Endocannabinoid hydrolysis generates brain prostaglandins that promote neuroinflammation. Science.

[88] Chen P., Hu S., Harmon S.D., Moore S.A., Spector A.A., Fang X. (2004). Metabolism of anandamide in cerebral microvascular endothelial cells. Prostag Other Lipid Mediat.

[89] Krivoshein G., Della Pietra A., Savinainen J., van den Maagdenberg A., Giniatullin R. (2025). Differential inhibitory effects of endocannabinoids on neuronal firing of mouse meningeal afferents. J Headache Pain.

[90] Mangutov E., Awad-Igbaria Y., Siegersma K., Gastambide F., Asuni A.A., Pradhan A.A.A. (2025). Enhancement of the endocannabinoid system through monoacylglycerol lipase inhibition relieves migraine-associated pain in mice. J Headache Pain.

[91] Liktor-Busa E., Levine A.A., Palomino S.M., Singh S., Wahl J., Vanderah T.W. (2023). ABHD6 and MAGL control 2-AG levels in the PAG and allodynia in a CSD-induced periorbital model of headache. Front Pain Res (Lausanne).

[92] Lau B.K., Drew G.M., Mitchell V.A., Vaughan C.W. (2014). Endocannabinoid modulation by FAAH and monoacylglycerol lipase within the analgesic circuitry of the periaqueductal grey. Br J Pharmacol.

[93] Dinh T.P., Carpenter D., Leslie F.M., Freund T.F., Katona I., Sensi S.L. (2002). Brain monoglyceride lipase participating in endocannabinoid inactivation. Proc Natl Acad Sci USA.

[94] Ramikie T.S., Patel S. (2012). Endocannabinoid signaling in the amygdala: anatomy, synaptic signaling, behavior, and adaptations to stress. Neuroscience.

[95] Egertova M., Cravatt B.F., Elphick M.R. (2003). Comparative analysis of fatty acid amide hydrolase and cb(1) cannabinoid receptor expression in the mouse brain: evidence of a widespread role for fatty acid amide hydrolase in regulation of endocannabinoid signaling. Neuroscience.

[96] Kano M., Ohno-Shosaku T., Hashimotodani Y., Uchigashima M., Watanabe M. (2009). Endocannabinoid-mediated control of synaptic transmission. Physiol Rev.

[97] Kuburas A., Russo A.F. (2023). Shared and independent roles of CGRP and PACAP in migraine pathophysiology. J Headache Pain.

[98] Csati A., Tajti J., Kuris A., Tuka B., Edvinsson L., Warfvinge K. (2012). Distribution of vasoactive intestinal peptide, pituitary adenylate cyclase-activating peptide, nitric oxide synthase, and their receptors in human and rat sphenopalatine ganglion. Neuroscience.

[99] Della Pietra A., Kuburas A., Russo A.F. (2025). PACAP versus CGRP in migraine: from mouse models to clinical translation. Cephalalgia.

[100] Gouveia-Figueira S., Goldin K., Hashemian S.A., Lindberg A., Persson M., Nording M.L. (2017). Plasma levels of the endocannabinoid anandamide, related N-acylethanolamines and linoleic acid-derived oxylipins in patients with migraine. Prostaglandins Leukot Essent Fatty Acids.

[101] Central pain study for ABX-1431. https://www.clinicaltrials.gov/study/NCT03138421?intr=ABX-1431&amp;rank=3.

[102] Greco R., Demartini C., Zanaboni A.M., Tumelero E., Icco R., Sances G. (2021). Peripheral changes of endocannabinoid system components in episodic and chronic migraine patients: a pilot study. Cephalalgia.

[103] Iversen H.K., Olesen J., Tfelt-Hansen P. (1989). Intravenous nitroglycerin as an experimental model of vascular headache. Basic characteristics. Pain.

[104] Hanalioglu S., Taskiran-Sag A., Karatas H., Donmez-Demir B., Yilmaz-Ozcan S., Eren-Kocak E. (2022). Cortical spreading depression can be triggered by sensory stimulation in primed wild type mouse brain: a mechanistic insight to migraine aura generation. J Headache Pain.

[105] Demartini C., Greco R., Francavilla M., Zanaboni A.M., Tassorelli C. (2022). Modelling migraine-related features in the nitroglycerin animal model: trigeminal hyperalgesia is associated with affective status and motor behavior. Physiol Behav.

[106] Raboisson P., Dallel R. (2004). The orofacial formalin test. Neurosci Biobehav Rev.

[107] De Logu F., Landini L., Janal M.N., Li Puma S., De Cesaris F., Geppetti P. (2019). Migraine-provoking substances evoke periorbital allodynia in mice. J Headache Pain.

[108] Hillard C.J. (2015). The endocannabinoid signaling system in the CNS: a primer. Int Rev Neurobiol.

[109] Greco R., Demartini C., Zanaboni A.M., Tumelero E., Reggiani A., Misto A. (2020). FAAH inhibition as a preventive treatment for migraine: a pre-clinical study. Neurobiol Dis.

[110] Greco R., Demartini C., Zanaboni A.M., Francavilla M., Reggiani A., Realini N. (2022). Potentiation of endocannabinoids and other lipid amides prevents hyperalgesia and inflammation in a pre-clinical model of migraine. J Headache Pain.

[111] Greco R., Bandiera T., Mangione A.S., Demartini C., Siani F., Nappi G. (2015). Effects of peripheral FAAH blockade on NTG-induced hyperalgesia--evaluation of URB937 in an animal model of migraine. Cephalalgia.

[112] Greco R., Demartini C., Zanaboni A., Casini I., De Icco R., Reggiani A. (2021). Characterization of the peripheral FAAH inhibitor, URB937, in animal models of acute and chronic migraine. Neurobiol Dis.

[113] Greco R., Demartini C., Zanaboni A.M., Berliocchi L., Piomelli D., Tassorelli C. (2018). Inhibition of monoacylglycerol lipase: another signalling pathway for potential therapeutic targets in migraine?. Cephalalgia.

[114] Nozaki C., Markert A., Zimmer A. (2015). Inhibition of FAAH reduces nitroglycerin-induced migraine-like pain and trigeminal neuronal hyperactivity in mice. Eur Neuropsychopharmacol.

[115] Imperatore R., Morello G., Luongo L., Taschler U., Romano R., De Gregorio D. (2015). Genetic deletion of monoacylglycerol lipase leads to impaired cannabinoid receptor CB(1)R signaling and anxiety-like behavior. J Neurochem.

[116] Schlosburg J.E., Blankman J.L., Long J.Z., Nomura D.K., Pan B., Kinsey S.G. (2010). Chronic monoacylglycerol lipase blockade causes functional antagonism of the endocannabinoid system. Nat Neurosci.

[117] Hillard C.J. (2000). Biochemistry and pharmacology of the endocannabinoids arachidonylethanolamide and 2-arachidonylglycerol. Prostag Other Lipid Mediat.

[118] Lichtman A.H., Martin B.R. (2005). Cannabinoid tolerance and dependence. Handb Exp Pharmacol.

[119] Aaltonen N., Savinainen J.R., Ribas C.R., Ronkko J., Kuusisto A., Korhonen J. (2013). Piperazine and piperidine triazole ureas as ultrapotent and highly selective inhibitors of monoacylglycerol lipase. Chem Biol.

[120] Greco R., Demartini C., Francavilla M., Zanaboni A.M., Tassorelli C. (2021). Dual inhibition of FAAH and MAGL counteracts migraine-like pain and behavior in an animal model of migraine. Cells.

[121] Akerman S., Holland P.R., Goadsby P.J. (2007). Cannabinoid (CB1) receptor activation inhibits trigeminovascular neurons. J Pharmacol Exp Therapeut.

[122] Fischer M.J., Messlinger K. (2007). Cannabinoid and vanilloid effects of R(+)-methanandamide in the hemisected meningeal preparation. Cephalalgia.

[123] Christiansen I.M., Edvinsson J.C.A., Reducha P.V., Edvinsson L., Haanes K.A. (2022). Dual action of the cannabinoid receptor 1 ligand arachidonyl-2'-chloroethylamide on calcitonin gene-related peptide release. J Headache Pain.

[124] Sarchielli P., Pini L.A., Coppola F., Rossi C., Baldi A., Mancini M.L. (2007). Endocannabinoids in chronic migraine: CSF findings suggest a system failure. Neuropsychopharmacology.

[125] Pamplona F.A., Ferreira J., Menezes de Lima O., Duarte F.S., Bento A.F., Forner S. (2012). Anti-inflammatory lipoxin A4 is an endogenous allosteric enhancer of CB1 cannabinoid receptor. Proc Natl Acad Sci USA.

[126] Vallee M., Vitiello S., Bellocchio L., Hebert-Chatelain E., Monlezun S., Martin-Garcia E. (2014). Pregnenolone can protect the brain from cannabis intoxication. Science.

[127] Kendall D.A., Yudowski G.A. (2016). Cannabinoid receptors in the central nervous system: their signaling and roles in disease. Front Cell Neurosci.

[128] Malan P.T., Ibrahim M.M., Deng H., Liu Q., Mata H.P., Vanderah T. (2001). CB2 cannabinoid receptor-mediated peripheral antinociception. Pain.

[129] Romero T.R., Resende L.C., Guzzo L.S., Duarte I.D. (2013). CB1 and CB2 cannabinoid receptor agonists induce peripheral antinociception by activation of the endogenous noradrenergic system. Anesth Analg.

[130] Greco R., Mangione A.S., Sandrini G., Nappi G., Tassorelli C. (2014). Activation of CB2 receptors as a potential therapeutic target for migraine: evaluation in an animal model. J Headache Pain.

[131] Christiansen I.M., Reducha P.V., Edvinsson L., Holm A., Haanes K.A. (2025). Ex vivo stimulation of the trigeminal nucleus caudalis induces peripheral CGRP release in the trigeminal ganglion and reveals a distinct dopamine-endocannabinoid mechanism relevant to migraine. J Headache Pain.

[132] Giuffrida A., Parsons L.H., Kerr T.M., Rodriguez de Fonseca F., Navarro M., Piomelli D. (1999). Dopamine activation of endogenous cannabinoid signaling in dorsal striatum. Nat Neurosci.

[133] Solinas M., Tanda G., Wertheim C.E., Goldberg S.R. (2010). Dopaminergic augmentation of delta-9-tetrahydrocannabinol (THC) discrimination: possible involvement of D(2)-induced formation of anandamide. Psychopharmacology (Berl).

[134] Lin Y.F. (2021). Potassium channels as molecular targets of endocannabinoids. Channels (Austin).

[135] Eroli F., Loonen I.C.M., van den Maagdenberg A., Tolner E.A., Nistri A. (2018). Differential neuromodulatory role of endocannabinoids in the rodent trigeminal sensory ganglion and cerebral cortex relevant to pain processing. Neuropharmacology.

[136] Ghovanloo M.R., Shuart N.G., Mezeyova J., Dean R.A., Ruben P.C., Goodchild S.J. (2018). Inhibitory effects of cannabidiol on voltage-dependent sodium currents. J Biol Chem.

[137] Ghovanloo M.R., Ruben P.C. (2022). Cannabidiol and sodium channel pharmacology: general overview, mechanism, and clinical implications. Neuroscientist.

[138] Benkli B., Kim S.Y., Koike N., Han C., Tran C.K., Silva E. (2023). Circadian features of cluster headache and migraine: a systematic review, meta-analysis, and genetic analysis. Neurology.

[139] Fuss J., Steinle J., Bindila L., Auer M.K., Kirchherr H., Lutz B. (2015). A runner's high depends on cannabinoid receptors in mice. Proc Natl Acad Sci USA.

[140] Tagliamonte S., Laiola M., Ferracane R., Vitale M., Gallo M.A., Meslier V. (2021). Mediterranean diet consumption affects the endocannabinoid system in overweight and obese subjects: possible links with gut microbiome, insulin resistance and inflammation. Eur J Nutr.

[141] Seng E.K., Martin P.R., Houle T.T. (2022). Lifestyle factors and migraine. Lancet Neurol.

[142] Acuna-Goycolea C., Obrietan K., van den Pol A.N. (2010). Cannabinoids excite circadian clock neurons. J Neurosci.

[143] Vaughn L.K., Denning G., Stuhr K.L., de Wit H., Hill M.N., Hillard C.J. (2010). Endocannabinoid signalling: has it got rhythm?. Br J Pharmacol.

[144] Valenti M., Vigano D., Casico M.G., Rubino T., Steardo L., Parolaro D. (2004). Differential diurnal variations of anandamide and 2-arachidonoyl-glycerol levels in rat brain. Cell Mol Life Sci.

[145] Hanlon E.C., Tasali E., Leproult R., Stuhr K.L., Doncheck E., de Wit H. (2015). Circadian rhythm of circulating levels of the endocannabinoid 2-arachidonoylglycerol. J Clin Endocrinol Metab.

[146] Perez-Morales M., De La Herran-Arita A.K., Mendez-Diaz M., Ruiz-Contreras A.E., Drucker-Colin R., Prospero-Garcia O. (2013). 2-AG into the lateral hypothalamus increases REM sleep and cFos expression in melanin concentrating hormone neurons in rats. Pharmacol Biochem Behav.

[147] Low Z.X.B., Lee X.R., Soga T., Goh B.H., Alex D., Kumari Y. (2023). Cannabinoids: emerging sleep modulator. Biomed Pharmacother.

[148] Poulsen A.H., Younis S., Thuraiaiyah J., Ashina M. (2021). The chronobiology of migraine: a systematic review. J Headache Pain.

[149] Kelman L., Rains J.C. (2005). Headache and sleep: examination of sleep patterns and complaints in a large clinical sample of migraineurs. Headache.

[150] Xie L., Kang H., Xu Q., Chen M.J., Liao Y., Thiyagarajan M. (2013). Sleep drives metabolite clearance from the adult brain. Science.

[151] Xie J.F., Wang L.X., Ren W.T., Wang C., Gao J.X., Chen H.L. (2023). An alpha-hemoglobin-derived peptide (m)VD-hemopressin (alpha) promotes NREM sleep via the CB(1) cannabinoid receptor. Front Pharmacol.

[152] Martin S.C., Gay S.M., Armstrong M.L., Pazhayam N.M., Reisdorph N., Diering G.H. (2022). Tonic endocannabinoid signaling supports sleep through development in both sexes. Sleep.

[153] Pava M.J., den Hartog C.R., Blanco-Centurion C., Shiromani P.J., Woodward J.J. (2014). Endocannabinoid modulation of cortical up-states and NREM sleep. PLoS One.

[154] Sun E., Motolani A., Campos L., Lu T. (2022). The pivotal role of NF-kB in the pathogenesis and therapeutics of Alzheimer's disease. Int J Mol Sci.

[155] Yuan W., Lu L., Rao M., Huang Y., Liu C.E., Liu S. (2021). GFAP hyperpalmitoylation exacerbates astrogliosis and neurodegenerative pathology in PPT1-deficient mice. Proc Natl Acad Sci USA.

[156] Rostami J., Mothes T., Kolahdouzan M., Eriksson O., Moslem M., Bergstrom J. (2021). Crosstalk between astrocytes and microglia results in increased degradation of alpha-synuclein and amyloid-beta aggregates. J Neuroinflammation.

[157] Cai X., Sun W., Cai M., Li D., Chen Z., Li H. (2025). Impaired glymphatic function contributes to high-frequency attacks in patients with episodic migraine. J Headache Pain.

[158] Burgos A.F., Olson P.A., Vgontzas A. (2024). The glymphatic system and its relationship to migraine. Curr Neurol Neurosci Rep.

[159] Kaag Rasmussen M., Mollgard K., Bork P.A.R., Weikop P., Esmail T., Drici L. (2024). Trigeminal ganglion neurons are directly activated by influx of CSF solutes in a migraine model. Science.

[160] Troili F., Cipollini V., Moci M., Morena E., Palotai M., Rinaldi V. (2020). Perivascular unit: this must be the place. The anatomical crossroad between the immune, vascular and nervous system. Front Neuroanat.

[161] Simard M., Arcuino G., Takano T., Liu Q.S., Nedergaard M. (2003). Signaling at the gliovascular interface. J Neurosci.

[162] Jessen N.A., Munk A.S., Lundgaard I., Nedergaard M. (2015). The glymphatic system: a beginner's guide. Neurochem Res.

[163] Navarrete M., Diez A., Araque A. (2014). Astrocytes in endocannabinoid signalling. Philos Trans R Soc Lond B Biol Sci.

[164] Moreno-Garcia A., Bernal-Chico A., Colomer T., Rodriguez-Antiguedad A., Matute C., Mato S. (2020). Gene expression analysis of astrocyte and microglia endocannabinoid signaling during autoimmune demyelination. Biomolecules.

[165] Bedse G., Bluett R.J., Patrick T.A., Romness N.K., Gaulden A.D., Kingsley P.J. (2018). Therapeutic endocannabinoid augmentation for mood and anxiety disorders: comparative profiling of FAAH, MAGL and dual inhibitors. Transl Psychiatry.

[166] Covelo A., Eraso-Pichot A., Fernandez-Moncada I., Serrat R., Marsicano G. (2021). CB1R-dependent regulation of astrocyte physiology and astrocyte-neuron interactions. Neuropharmacology.

[167] Krishnan K.S., Billups B. (2023). ASC transporters mediate D-Serine transport into astrocytes adjacent to synapses in the mouse brain. Biomolecules.

[168] Camberos-Barraza J., Camacho-Zamora A., Batiz-Beltran J.C., Osuna-Ramos J.F., Rabago-Monzon A.R., Valdez-Flores M.A. (2024). Sleep, glial function, and the endocannabinoid system: implications for neuroinflammation and sleep disorders. Int J Mol Sci.

[169] Mestre L., Docagne F., Correa F., Loria F., Hernangomez M., Borrell J. (2009). A cannabinoid agonist interferes with the progression of a chronic model of multiple sclerosis by downregulating adhesion molecules. Mol Cell Neurosci.

[170] Amenta P.S., Jallo J.I., Tuma R.F., Elliott M.B. (2012). A cannabinoid type 2 receptor agonist attenuates blood-brain barrier damage and neurodegeneration in a murine model of traumatic brain injury. J Neurosci Res.

[171] Amenta P.S., Jallo J.I., Tuma R.F., Hooper D.C., Elliott M.B. (2014). Cannabinoid receptor type-2 stimulation, blockade, and deletion alter the vascular inflammatory responses to traumatic brain injury. J Neuroinflammation.

[172] Piantino J., Lim M.M., Newgard C.D., Iliff J. (2019). Linking traumatic brain injury, sleep disruption and post-traumatic headache: a potential role for glymphatic pathway dysfunction. Curr Pain Headache Rep.

[173] Panikashvili D., Shein N.A., Mechoulam R., Trembovler V., Kohen R., Alexandrovich A. (2006). The endocannabinoid 2-AG protects the blood-brain barrier after closed head injury and inhibits mRNA expression of proinflammatory cytokines. Neurobiol Dis.

[174] Panikashvili D., Simeonidou C., Ben-Shabat S., Hanus L., Breuer A., Mechoulam R. (2001). An endogenous cannabinoid (2-AG) is neuroprotective after brain injury. Nature.

[175] Gruenbaum S.E., Zlotnik A., Gruenbaum B.F., Hersey D., Bilotta F. (2016). Pharmacologic neuroprotection for functional outcomes after traumatic brain injury: a systematic review of the clinical literature. CNS Drugs.

